# The Effect of Childhood Health Status on Adult Health in China

**DOI:** 10.3390/ijerph15020212

**Published:** 2018-01-26

**Authors:** Qing Wang, Huyang Zhang, John A. Rizzo, Hai Fang

**Affiliations:** 1 School of Business, Dalian University of Technology, Panjin 124221, China; wangqing1984@126.com; 2 China Center for Health Development Studies, Peking University, Beijing 100083, China; sunshineclan@yeah.net; 3 Department of Health Policy and Administration, Peking University, Beijing 100083, China; 4 Department of Family, Population & Preventive Medicine, State University of New York at Stony Brook, Stony Brook, NY 11790, USA; John.Rizzo@stonybrookmedicine.edu

**Keywords:** childhood health, adult health, adult cognition, adult physical function, China

## Abstract

Childhood health in China was poor in the 1950s and 1960s because of limited nutrition. In the last three decades, China has distinguished itself through its tremendous economic growth and improvements in health and nutrition. However, prior to such growth, access to good nutrition was more variable, with potentially important implications, not only for childhood health, but also for adult health, because of its long-term effects lasting into adulthood. To shed light on these issues, this study examined the long-run association between childhood health and adult health outcomes among a middle-aged Chinese population and addresses the endogeneity of childhood health. A nationwide database from the 2011 China Health and Retirement Longitudinal Study (CHARLS) was employed. Three adult health outcomes variables were used: self-reported health status, cognition, and physical function. The local variation in grain production in the subjects’ fetal period and the first 24 months following birth was employed as an instrument for childhood health in order to correct for its endogeneity. Childhood health recalled by the respondents was positively and significantly associated with their adult health outcomes in terms of self-reported health status, cognition, and physical function in single-equation estimates that did not correct for the endogeneity of childhood health. A good childhood health status increased the probabilities of good adult health, good adult cognitive function, and good adult physical function by 16% (95% CI: 13–18%), 13% (95% CI: 10–15%), and 14% (95% CI: 12–17%), respectively. After correcting for endogeneity, the estimated effects of good childhood health were consistent but stronger. We also studied the male and female populations separately, finding that the positive effects of childhood health on adult health were larger for males. In China, childhood health significantly affects adult health. This suggests that early interventions to promote childhood health will have long-term benefits in China and that health-care policies should consider their long-term impacts over the life cycle in addition to their effects on specific age groups.

## 1. Introduction

Individual health is not static and often changes as a result of the exposure to various life events [1,2]. Previous research shows that a poor childhood health status has a long-range negative influence on adult health outcomes in terms of self-reported health status, chronic diseases, and successful aging [3,4,5,6,7,8,9,10,11,12,13]. However, the majority of these studies are from developed countries including the United States, France, and the United Kingdom. Little is known about this issue in developing countries, and the results are inconsistent. Grimard concluded that a better childhood health up to 10 years of age positively affected adult health outcomes in Mexico [14]. McEniry suggested that unfavorable conditions (e.g., poor nutrition and infectious diseases) in utero were related to heart disease and diabetes, but found no association with functionality and poor health at an older age among a Puerto Rican population [15,16]. Margolis found that Guatemalan adults who suffered from high levels of morbidity during childhood were more likely to report poor health, even adjusting for family characteristics, health behaviors, and adult socioeconomic status. However, diarrheal disease at older age was found to be related to lower levels of triglycerides and plasma glucose [17].

Chinese disease profiles and welfare state institutions differ greatly from other countries. Malnutrition during the 1950s and 1960s significantly influenced childhood health status in China. However, in the past 30 years, life expectancy in China more than doubled from 35 to 76 years, largely because of China’s rapid and sustained economic development [18]. The question of whether and to what extent poor childhood nutrition and health in the 1950s and 1960s has affected adult health outcomes in China remains unclear. Quantifying this relationship is important for understanding how health and nutrition policies affect health outcomes over the entire life cycle. Chen and Zhou observed unfavorable impacts of the great famine of 1959–1962 on adult health in China, hypothesizing that a poor nutrition adversely affected child health, eventually leading to poor adult health [19]. Song also studied the intergenerational correlation of the great famine and infant mortality [20]. Ma analyzed the long-term relationship between socioeconomic status and obesity from childhood through adulthood [21]. Smith observed significant effects of childhood health on adult health outcomes for Chinese women and on adult Body Mass Index (BMI), particularly among Chinese men, but did not consider the endogeneity of childhood health [22,23]. To the best of our knowledge, no study has quantified the long-term effects of childhood health on adult health on the basis of nationwide data from China [15].

The present study seeks to address this issue by estimating the effects of childhood health on adult health outcomes in people between 45 and 61 years of age in 2011. This has important public health implications. If childhood health is found to be associated with health in adulthood in China, then public health interventions focusing on childhood health will lead to additional long-range benefits.

## 2. Data and Methods

### 2.1. Data: The China Health and Retirement Longitudinal Study (CHARLS)

Data were obtained from the China Health and Retirement Longitudinal Study (CHARLS) 2011. The CHARLS is a nation-representative sample of Chinese middle-aged and elderly persons aged 45 and above. More information about the CHARLS data can be found at http://charls.ccer.edu.cn/en. The baseline national wave of CHARLS was conducted in 2011 and covered approximately 10,000 households among 150 counties and districts. The overall response rate was 85%. The full sample consists of 17,500 subjects with a mean age of 59.05 (the standard deviation is 10.15). The age distribution in the CHARLS database in 2011 is very similar to that in the 2010 China census. A multi-stage stratified Probability-Proportionate-to-Size sampling framework was applied. Following the Health and Retirement Study in the United States, the English Longitudinal Study of Aging in the United Kingdom, and the Survey of Health, Aging and Retirement in Europe, the CHARLS collected various characteristics, including demographics, family structure and transfer, childhood and adult socioeconomic status, health status and functioning, and health behaviors. The present study only includes respondents born after 1949, because data on the fluctuations in grain production used as an instrument variable in the present study was unavailable prior to 1949. After excluding some observations with missing values and being born during World War II and the civil war in China (1946–1949), the final sample size was 7589. CHARLS is an open-access database, which is approved by the Institutional Review Board of Peking University.

### 2.2. Adult Health Outcomes

Three adult health outcomes variables were used in this study: self-reported health status, cognition, and physical function. The self-reported health status was based on respondents’ answers to the question: “Would you say your health is excellent, very good, good, fair, or poor?”, and a binary variable was constructed to rank the self-reported health statuses as 0 (poor, or fair) or 1 (good, very good, or excellent).

Since our study focused on middle-aged persons, and high mental and physical functioning have been recognized as predictors of “successful aging” in previous literature, we also used measures of cognition and physical function as our outcome variables [24]. We followed Brandt et al. to measure good cognition and physical function [9]. A respondent was defined as having good cognition if he or she scored a median or higher score on a cognitive functioning index including the following questions: naming correctly the date, the week, the month, season, and year (1 point for each correct answer and maximum point score of 5); an immediate 10-word recall test and a delayed 10-word recall test (1 point for each correctly recalled word and maximum point score of 20); a mathematical performance test (1 point for each correct answer and maximum point score of 5) [25]. The participants could obtain a maximum overall score of 30. The median score in the CHARLS 2011 sample was 11. The respondents’ cognition was defined as good (equal to 1) if his or her score was higher than or equal to 11, otherwise it was classified as 0, indicating poor cognition [9].

Following Brandt et al., a respondent was regarded as having physical functional deficits if he or she had difficulties in more than one of the following thirteen activities: running or jogging 1000 m; stooping, kneeling, or crouching; getting up from a chair after sitting for a long period; reaching or extending arms above shoulder level; climbing several flights of stairs; lifting or carrying items weighing more than 5 kg; picking up a small coin from a table; dressing; bathing; eating; getting into or out of bed; using the toilet; controlling urination and defecation [9]. This physical function variable equaled one (good physical function) if the respondent reported difficulties performing no more than one of these activities and equaled zero otherwise.

### 2.3. Childhood Health and Other Control Variables

Childhood health was estimated through the following question: “How would you evaluate your health during childhood, up to and including age 15: excellent, very good, good, fair, or poor?”, and a category variable of “good childhood health” was constructed. If the respondent reported his or her childhood health as “excellent”, “very good”, or “good”, the “good childhood health” variable was encoded as 1, otherwise it was scored 0.

As noted earlier, previous studies have shown that childhood socioeconomic status affects health at later ages, so variables of childhood socioeconomic status should also be considered [14,26,27]. Childhood socioeconomic status was evaluated by (a) the father’s education and (b) the number of siblings in the household. The father’s education was categorized by three levels: junior high school or below, senior high school, and college or above. Two binary variables were constructed to reflect the education levels of senior high school and college or above, respectively, while the education level of junior high school or below (which had the highest frequency among the respondents) served as the reference group. During the period of China’s planned economy (before 1978), household incomes were very similar for all households within the same geographic area. Thus, the more children a household had, the fewer resources each household member could receive. The subjects studied in this paper were born between 1949 and 1966, a period during which fertility was not well controlled [28]. During this time period, the population control policies varied, but in general family planning was not enforced, and contraceptive methods were limited. Most families had no access to birth control, and the social perception regarding birth control was negative. The total fertility rates in the 1950s and 1960s averaged approximately 6 children per woman, so most children in our study had at least one sibling. We controlled for the number of siblings in the household, since more siblings in one household means fewer resources for each child [29]. The period of the Great famine (1959–1962) was included as a negative health shock. More specifically, a binary variable equal to 0 if the individual was exposed to the great famine in utero and up to age 2, and otherwise, 0. The famine cohort was the reference group. Early-life local conditions were considered by including provinces’ fixed effects (birth province dummy variables).

Variables such as respondents’ current socioeconomic status and health behaviors were important determinants of adult health outcomes and were also included in the models. The adult educational attainment measuring respondents’ current socioeconomic status was classified into three levels: junior high school or below, senior high school, and college or above. Two binary variables were constructed, with junior high school or below serving as the reference group. Whether the respondent was a current smoker, a drinker, or both were controlled to measure respondents’ current health behaviors. Demographic variables such as gender (reference group: female), marital status (reference group: married with spouse present (common-law marriage was considered as married), and age were also included. The respondents were categorized into the following groups: aged 45–50; aged 50–55; aged 55–60; aged 60+. The cohort aged 60+ served as the reference group. Since genetic factors are obviously important determinants of health, a binary variable indicating whether the respondent’s father lived beyond 65 years of age was included as a rough proxy for genetic factors.

### 2.4. Estimation Strategy

We used logistic regression models to estimate the association between childhood health status and adult health outcomes, adjusting for childhood socioeconomic status, respondents’ current socioeconomic status, respondents’ current health behaviors, and demographic variables:

(1) $$H_{adult}=\beta_{0}+\beta_{1}H_{child}+\beta_{2}X+\varepsilon$$

where $H_{adult}$ = adult health outcome; $H_{child}$ = childhood health status before 15 years of age; X = a vector for other control variables; ε = a disturbance term; and $\beta_{0}\beta_{1}\beta_{2}$ = coefficients to be estimated.

**Figure A1:**
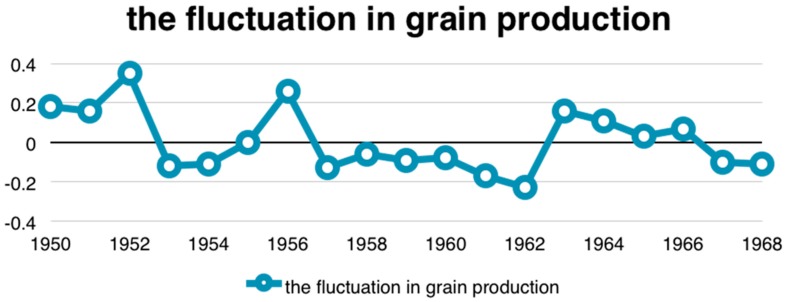
The fluctuation in grain production by year.

An endogeneity issue of children health could occur due to a measurement error, a simultaneous causality, or omitted variables (please see Appendix A.1) [30,31]. In order to address these endogeneity problems, this study employed the two-stage residual inclusion estimation approach (2SRI) with a valid instrument variable [32,33]. The instrument used in this study was the fluctuation in grain production in the subject’s residential province during the subjects’ fetal period (about 9 months) and for the first 24 months following birth (a total of 33 months). That is, the instrument was the deviation from trend in grain production, which is the ratio of the actual grain yield divided by the average trend of grain yield. The average trend of grain yield was calculated according to the HP (Hodrick–Prescott) filter method predicting grain yields using data from the China National Statistics Yearbook from 1949 to 2008 (please see Appendix A.2) [34]. The instrumental variable ranged from −0.24 to 0.28 in this study, and its mean was −0.005. The grain production levels varied substantially across geographic areas during the period 1949–1968 (see Appendix B Figure A1).

The fluctuation in grain production is an indicator that measures the availability of grain production. As grain production in the birth area increases during the respondent’s fetal period and the first two years of life, childhood nutrition should improve [16]. Nutrition during pregnancy and infancy has been found to affect childhood health significantly [35,36]. As expected, the grain production within the respondent’s fetal period and the first two years of life in the birth area was positively correlated with his or her childhood health status (see Appendix B Table A2).

Fluctuations in grain production during the respondent’s fetal period and the first two years of life in the birth area did not directly affect adult health outcomes. The well-known Dutch famine studies have indicated an unfavorable influence of malnutrition on childhood health, but no long-range consequences for adult health [37,38]. In addition, the present study performed a number of statistical tests of the instrumental variable (i.e., the excluded instrument test, underidentification test, weak identification test, and weak-instrument robust inference test) that demonstrated that the instrument was valid (see Appendix B Table A3).

Since all the dependent variables are binary, we used the Two-Stage Residual Inclusion (2SRI) Estimation. The 2SRI estimation has been shown to yield consistent and efficient results, so that it is widely used to solve endogeneity issues in studies where the second-stage dependent variable is binary [31,38,39,40,41].

The first-stage equation was estimated as follows:

(2) $$H_{child}=\alpha_{0}+\alpha_{1}IV+\alpha_{2}X+u$$

where IV = instrumental variables; u = residual; $\alpha_{0}\alpha_{1}\alpha_{2}$ = coefficients to be estimated.

The second-stage equation could be estimated by inserting the residual from the first stage into Equation (1) as follows:

(3) $$H_{child}=\beta_{0}+\beta_{1}IV+\beta_{2}X+\beta_{3}\hat{u}+\varepsilon$$

$\beta_{0}\beta_{1}\beta_{2}$ = coefficients to be estimated in the second stage;

$\hat{u}$ = the predicted residual in the first stage;

ε = residual in the second stage.

Since the childhood self-reported health in the first stage is a category variable, logistic regression was used, and the generalized residual from the logistic model in the first stage was added into the second stage [39]. The standard errors produced from the nonlinear system using this approach were incorrect, however, as they failed to account for the stochastic nature of the estimated residual terms. Therefore, bootstrapping with 1000 iterations was used to correct the standard errors [40]. All statistical analyses in the paper were performed using Stata 12 (StataCorp. LLC, College Station, TX, USA).

## 3. Results

Table 1 shows descriptive statistics by gender. Three percent of the respondents reported their current health status as excellent; 37% as very good; 24% as good; 10% as fair; and 26% as poor. In addition, the cognition of 43% of the respondents was found to be good, while 45% of the respondents had good physical function. A total of 65% of respondents reported their childhood health as excellent, very good, or good. Males were more likely to report poor adult health outcomes measured by self-report health, cognition, and physical function than females, but these differences are statistically insignificant.

The effects of childhood health on adult health are presented in Table 2, after adjusting for childhood socioeconomic status, current socioeconomic status, health behaviors, and demographic characters. Childhood health status was related to good adult health, good adult cognition, and good adult physical function. Compared to respondents with poor childhood health, in the logistic regression without addressing endogeneity, the odds ratio of good adult self-reported health, good adult cognitive function, and good adult physical function increased by 1.95 (95% CI: 1.73–2.20), 1.74 (95% CI: 1.55–1.96), and 1.84 (95% CI: 1.64–2.07), respectively. The results were similar to the regression results without controlling for child factors (see Appendix B Table A1). Moreover, the results were very robust when we expanded our samples to all respondents above 45 (including respondents who were born before 1949), excluded individuals who were born during the years 1959–1962, when the great famine occurred, or excluded 859 respondents who mainly lived in cities or towns before they were 16 years old. Because of manuscript length constraints, such results are not present in the manuscript, but available from the authors upon request [19,41].

When estimated with 2SRI, the effects of childhood health were consistent, and the residual added into the second stage was significant as well, implying that childhood health status is endogenous. The effects calculated from 2SRI were larger than those estimated without correcting for endogeneity. However, the very large odds ratio change for the association of childhood health and adult health between the logistic regression (1.95) and the 2SRI (37.67) may be due to overestimation.

Table 3 presents the marginal effects of child health status on adult health outcomes. In the multivariate logistic regression results without correcting for endogeneity we found that: a good childhood health status increased the probability of good self-reported adult health, good adult cognitive function, and good adult physical function by 16% (95% CI: 13–18%), 13% (95% CI: 10–15%), and 14% (95% CI: 12–17%), respectively. When estimated with 2SRI, the marginal effects of childhood health status on adult health outcomes increased. A good childhood health status increased the likelihood of good self-reported adult health, good adult cognitive function, and good adult physical function by 60% (95% CI: 56–65%), 38% (95% CI: 31–44%), and 43% (95% CI: 37–51%), respectively. A possible explanation for the difference between the endogeneity-corrected and uncorrected results is that the respondents with good adult health tended to overreport that their childhood health was good, leading to weaker estimated effects of childhood health on adult health in the models that do not correct for endogeneity [14]. The marginal effect changes of adult self-reported health, cognition, and physician function from the logistic model to the 2SRI model were similar and consistent.

Table 4 presents the results stratified by gender using the 2SRI and logistic models. Because of space limitations, only the odds ratios of childhood health and residuals in the 2SRI model are reported and other control variables are omitted (the full set of results is available from the authors upon request). Childhood health affected males’ health measured by self-reported health, cognition, and physical function at a later age, which is consistent with the whole sample. However, for females, childhood health significantly influenced adult physical function, but not self-reported health and cognition.

## 4. Discussion

Using the CHARLS 2011 database, this study estimated the long-run impacts of childhood health on health outcomes at an older age in China. We observed a strong relationship between childhood health and elders’ likelihood of having good health outcomes measured by self-reported health, cognition, and physical function in China, even after adjusting for demographic, adult characteristics, and family background. In order to address the endogeneity of childhood health status, we employed an instrumental variable (the fluctuation in grain production in the birth area during the fetal period and two years after the birth month) and the 2SRI estimation. A set of tests supported that the instrument was valid, and the fluctuation of grain production in the birth district in the fetal period and two years after the birth month was positively and significantly related to childhood health status. The results of the 2SRI estimation indicated that the effects of childhood health on health at an older age were positive, but larger than the results without adjusting for endogeneity. In China, even though the respondents may have tended to overrate their childhood health because of their current health status, we did observe that childhood health was associated with health at an older age.

Our results are consistent with most of the studies in the previous literature, but in contrast with the results about the relationship between childhood health and physical function and self-reported health described in a study using data from Puerto Rico [16]. The heterogeneous results may be explained by the pathway linking childhood health with adult health. According to the critical period programming model, children in utero, at birth, or in early infancy may adapt to malnutrition conditions by developing “thrifty genes” [42,43]. Although “thrifty genes” may adjust the body’s physiology and metabolism to enhance survival, they may induce higher risks of obesity, type 2 diabetes, and coronary heart diseases if such children enjoy good nutrition later in life [42]. In this case, poor nutrition is not followed by good nutrition, the critical period programming model may not predict a relationship between childhood health and adult health. With the rapid economic growth in China, adults exposed to severe malnutrition are now experiencing higher dietary energy intakes [44]. As a result, such individuals may be more likely to report poor health. In addition, “thrifty genes” may be more closely related to suffering from obesity, type 2 diabetes, and coronary heart diseases rather than other health outcomes.

Childhood health had a stronger effect on adult health for males than for females, consistent with previous studies in China and Estonia, but at odds with a study in the United States [12,19,41,45]. A potential explanation consists in the mortality selection effects. Biologically, the mortality selection effects could be gender-biased, resulting in a long-term health disparity depending on gender. Males were observed to suffer higher excess mortality rates due to poor nutrition during difficult times in countries such as West Bengal, Denmark, and China [46,47]. In other words, females had a mortality advantage over their male counterparts [48,49,50]. On the other hand, male survivors seemed to be well endowed with some genetic or congenital traits that may have reduced health risks later in life because of a difficult tough selection process [50]. Therefore, the mortality selection hypothesis predicts better health outcomes for males as adults in China, while children in the United States did not experience such a mortality selection situation, thus, the stronger effects were observed for males in the United States.

This is the first study to quantify long-run effects of childhood health on adult health using nationwide data from China. While these results are consistent with growing evidence from developed and developing countries, they contribute to the literature in this field by providing evidence with relatively broad adult health indicators (e.g., self-reported health status, good cognition, and physical functions) for a previously understudied, yet growing, demographic population of middle-aged subjects in China. We also used sophisticated statistical techniques (i.e., two-stage residual inclusion estimation) to address endogeneity problems and obtain more precise effects of childhood health in the long run.

This study has several limitations that should be noted. First, our work on health in CHARLS relied on a self-evaluation health status, without taking other health measures such as chronic disease into account. Second, a measurement error or bias could exist in terms of self-evaluation health. In contrast to our expectation, we found that exposure to famine increased the likelihood of self-reported good health in older age. It may be that subjects not affected by the famine period had higher expectations for good health so that they self-reported a worse health status. Their objective health measures of cognitive and physical health were higher, however, which lends some credence to this possible interpretation.

Our results have several health policy implications for China. First, instead of focusing on the health of specific age groups, health care policies in China should consider their long-term impacts over the life cycle. For instance, the fact that childhood health matters to achieve successful aging, including avoidance of disease and disability, maintenance of high physical and cognitive function, and sustained engagement in social and productive activities, implies that interventions to improve elderly health should take into consideration the events in the life course, beginning in childhood. Understanding these long-term effects on health seems particularly salient given that life expectancy is expected to grow. In addition, adult health inequalities may partly result from health disparities during childhood. Consequently, policies aimed at decreasing health inequalities among the middle-aged and elderly population should also seek to reduce health disparities during childhood.

## 5. Conclusions

Using a nationally representative data, this study attempts to estimate the effects of childhood health on adult health. Childhood good health was related to adult good health measured by self-report overall health, cognition and physical function. Those born in the 1950s and 1960s were exposed to poor nutrition during childhood period, which may have enduring effects on health in later age. Thus, a life-course health intervention should be enforced, rather than targeting specfic age groups.

## Figures and Tables

**Table 1:** Descriptive statistics.

Variables	All Sample *n* = 7589	Male *n* = 4629	Female *n* = 2960
Adult health outcomes			
Self-reported health status (%)			
Excellent	3	3	3
Very good	37	36	39
Good	24	19	32
Fair	10	9	12
Poor	26	33	15
Good cognition (%)	43	36	54
Good physical Function (%)	45	37	58
Childhood health outcomes			
Self-reported health status up to and including age 15 (%)			
Excellent	5	5	5
Very good	34	28	43
Good	26	32	17
Fair	29	28	31
Poor	6	7	4
Fluctuation of grain production (mean and SD)	−0.005 (0.15)	−0.003 (0.13)	−0.008 (0.17)
Other control variables			
Education level (%)			
Junior high school or below	87	89	84
Senior high school	12	10	15
College or above	1	1	1
Current smoker (%)	27	26	29
Current drinker (%)	19	16	24
Not married (%)	12	11	14
Male (%)	61	100	0
Age (mean and SD)	53.60 (5.07)	54.05 (5.09)	52.90 (4.96)
Father’s education level (%)			
Junior high school or below	92	95	88
Senior high school	2	4	4
College or above	6	1	8
Number of siblings (mean and SD)	3.97 (1.67)	3.89 (1.56)	4.08 (1.82)
The father lived longer than 65 years (%)	11	8	16
Exposure to the great famine	60.73	61.78	59.09

Note: If the respondent’s self-reported health was “excellent”, “very good”, or “good”, the respondent was considered in good health.

**Table 2:** Effects of childhood health status on adult health outcomes.

Variable	Logistic Model			2SRI		
	Self-Reported Health Status	Good Cognition	Good Physical Function	Self-Reported Health Status	Good Cognition	Good Physical Function
	Odd Ratio (95% CI)	Odd Ratio (95% CI)	Odd Ratio (95% CI)	Odd Ratio (95% CI)	Odd Ratio (95% CI)	Odd Ratio (95% CI)
Good childhood health	1.95	1.74	1.84	37.67	5.32	7.45
	(1.73–2.20)	(1.55–1.96)	(1.64–2.07)	(24.88–57.03)	(3.60–7.87)	(5.00–11.12)
The residual of the first stage				0.20	0.54	0.46
				(0.16–0.25)	(0.44–0.67)	(0.37–0.58)
Education level (Junior high school or below serves as the reference group)						
Senior high school	0.70	1.54	1.65	0.60	1.44	1.53
	(0.59–0.83)	(1.30–1.81)	(1.40–1.95)	(0.51–0.71)	(1.22–1.70)	(1.29–1.81)
College or above	0.53	1.70	2.58	0.61	1.78	2.74
	(0.27–1.04)	(0.94–3.07)	(1.33–4.99)	(0.31–1.20)	(0.98–3.23)	(1.41–5.31)
Current smoker	0.69	1.97	2.00	0.40	1.58	1.52
	(0.60–0.79)	(1.73–2.23)	(1.76–2.27)	(0.34–0.46)	(1.36–1.83)	(1.31–1.76)
Current drinker	1.07	1.18	1.23	1.06	1.18	1.23
	(0.92–1.24)	(1.03–1.36)	(1.07–1.42)	(0.91–1.23)	(1.02–1.36)	(1.06–1.42)
Not married	0.75	2.18	2.36	0.39	1.69	1.71
	(0.63–0.89)	(1.85–2.57)	(2.00–2.79)	(0.32–0.47)	(1.40–2.03)	(1.42–2.07)
Male	0.91	0.53	0.54	1.16	0.58	0.61
	(0.82–1.02)	(0.48–0.59)	(0.48–0.60)	(1.04–1.30)	(0.52–0.65)	(0.54–0.68)
Aged 45–50	0.58	1.57	1.63	0.86	1.82	1.96
	(0.50–0.67)	(1.35–1.83)	(1.40–1.89)	(0.73–1.00)	(1.56–2.13)	(1.67–2.29)
Aged 50–55	0.36	1.00	0.80	1.11	1.55	1.38
	(0.31–0.43)	(0.85–1.19)	(0.68–0.95)	(0.88–1.40)	(1.23–1.95)	(1.10–1.74)
Aged 55–60	0.68	1.30	1.13	1.01	1.51	1.38
	(0.52–0.88)	(0.98–1.71)	(0.86–1.50)	(0.77–1.33)	(1.14–2.00)	(1.04–1.83)
Non-famine cohort	0.68	1.45	1.57	0.79	1.53	1.68
	(0.58–0.79)	(1.24–1.69)	(1.35–1.83)	(0.68–0.92)	(1.31–1.79)	(1.44–1.97)
Father education (Junior high school or below serves as the reference group)						
Senior high school	0.82	1.12	0.99	0.71	1.06	0.92
	(0.58–1.15)	(0.80–1.56)	(0.70–1.39)	(0.50–1.01)	(0.76–1.48)	(0.66–1.29)
College or above	1.08	1.58	1.43	1.17	1.63	1.49
	(0.86–1.35)	(1.25–1.99)	(1.14–1.80)	(0.94–1.47)	(1.29–2.05)	(1.19–1.87)
The log of number of siblings	0.64	1.19	1.22	0.76	1.27	1.32
	(0.57–0.72)	(1.06–1.34)	(1.08–1.37)	(0.67–0.86)	(1.13–1.43)	(1.17–1.49)
Father lived longer than 65 years	0.92	1.28	1.17	0.74	1.18	1.05
	(0.77–1.10)	(1.09–1.52)	(0.99–1.39)	(0.62–0.88)	(0.99–1.40)	(0.89–1.25)

Note: If the respondent’s self-reported health was “excellent”, “very good”, or “good”, the respondent was considered as having had good childhood health. Provinces' fixed effects were included.

**Table 3:** The marginal effects of good childhood health on adult health outcomes.

Adult Health Outcomes	Good Childhood Health Marginal Effects	
	Logistic Model Marginal Effects (95% CI)	2SRI Model Marginal Effects (95% CI)
Good self-reported health	0.16 (0.13–0.18)	0.60 (0.56–0.65)
Good cognition	0.13 (0.10–0.15)	0.38 (0.31–0.44)
Good physical function	0.14 (0.12–0.17)	0.43 (0.37–0.51)

**Table 4:** Effects of childhood health status on adult health outcomes by gender using 2SRI.

Estimation Strategy	Variables	Male			Female		
		Self-Reported Health Status	Good Cognition	Good Physical Function	Self-Reported Health Status	Good Cognition	Good Physical Function
		Odd Ratio (95% CI)	Odd Ratio (95% CI)	Odd Ratio (95% CI)	Odd Ratio (95% CI)	Odd Ratio (95% CI)	Odd Ratio (95% CI)
2SRI	Good childhood health	17.10	21.71	16.45	1.16	1.99	13.64
		(9.48–30.86)	(11.74–40.13)	(8.95–30.23)	(0.04–35.97)	(0.07–60.55)	(0.44–420.28)
	The residual of the first stage	0.41	0.34	0.37	0.87	0.69	0.27
		(0.31–0.54)	(0.25–0.47)	(0.27–0.51)	(0.14–5.58)	(0.11–4.35)	(0.04–1.74)
Logistic model	Good childhood health	2.83	2.67	2.39	0.89	0.99	1.24
		(2.32–3.43)	(2.21–3.22)	(1.98–2.88)	(0.76–1.05)	(0.84–1.17)	(1.05–1.46)

Note: If the respondent’s self-reported health was “excellent”, “very good”, or “good”, the respondent was considered as having had good childhood health. Demographic, current socioeconomic status, current health behaviors, childhood factors, and provinces' fixed effects were included.

## Abbreviations

BMI	Body Mass Index
CHARLS	The China Health and Retirement Longitudinal Study
2SRI	Two-Stage Residual Inclusion
2SLS	Two-Stage Least Squares

## Appendix A

### Appendix A.1. Potential Origins of Endogeneity: Measurement Error, Simultaneous Causality, and Omitted Variables Issues

Measurement error issues exist for our “self-reported adult and child health status”. If there are measurement errors in the dependent variable (adult health status), these measurement errors will not be correlated with the key explanatory variable (child health status), so the coefficient estimate on child health status is still unbiased [30]. However, if there are measurement errors in the key explanatory variable (child health status) an endogeneity issue arises [30]. Intuitively, if the explanatory variable (child health status) has a considerable measurement error, it will be a very noisy proxy for the actual child health status. In this case, the estimated effect of child health status on adult health status would be biased toward zero. In an extreme case, where the explanatory variable is purely random noise, the estimated coefficient on it would approach zero.

Furthermore, unobserved omitted variables and/or simultaneity can potentially lead to endogeneity [31]. We were unable to directly measure childhood health status; instead, we had to count on the respondents’ recollections about childhood health. Many factors such as the respondents’ socioeconomic status and demographic characteristics may impact the respondents’ recall responses about their childhood health; in addition, some of them are also unavailable in our database, resulting in omitted variables issues. Additionally, adult current health may have also impacted self-reported childhood health, resulting in a simultaneity problem.

### Appendix A.2. How Was the Instrumental Variable "Grain Production" Calculated?

The HP (Hodrick–Prescott) filter method was used to predict the average trend of grain yield. Grain production in the time series is usually characterized by “fluctuations in growth” or “fluctuations in the reduction”, and it is a combination of the long-term trend and short-term fluctuations. Therefore, The HP (Hodrick–Prescott) filter method was adopted to separate long-term trend and short-term fluctuations of grain output. The long-term trend sequence is relatively stable, and can be used for economic forecasting.

The Hodrick–Prescott filter separates the time series of grain production $y_{t}$ into a trend component $x_{t}$ and a cyclical component $c_{t}=y_{t}-x_{t}$ It is equivalent to a cubic spline smoother, with the smoothed portion in $x_{t}$.

The objective function for the filter has the form:

$$\underset{x_{t}}{\min}\sum_{t=1}^{T}\left\{{\left(y_{t}-x_{t}\right)}^{2}+\lambda \left[{\left(x_{t+1}-x_{t}\right)}^{2}+{\left(x_{t}-x_{t-1}\right)}^{2}\right]\right\}$$

where t is the number of samples and λ is the smoothing parameter,  λ=100. The programming problem is to minimize the objective over all $t_{1}$,…$t_{T}$,. The first sum minimizes the difference between the time series and its trend component (which is its cyclical component). The second sum minimizes the second-order difference of the trend component (which is analogous to minimization of the second derivative of the trend component). The analyses were performed in Stata 12, using the “tsfilter hp” command.

Individuals having different months of birth spent heterogeneous amounts of time in their fetal period and the first two years. For example, an individual born on 1 January 1958 had 10 months in 1957 for fetal period, and 12 months in 1958 and 12 months in 1959 for the first two years of his or her life. We therefore calculated the fluctuation in grain production relative to the trend in production during the respondent’s fetal period and the first two years of life in his or her birth area.

## Appendix B

**Table A1:** Impact of childhood health on adult health outcome without adjusting for child factors using the Logistic model.

Variable	Logistic Model			2SRI		
	Self-Reported Health Status	Good Cognition	Good Physical Function	Self-Reported Health Status	Good Cognition	Good Physical Function
	Odd Ratio	Odd Ratio	Odd Ratio	Odd Ratio	Odd Ratio	Odd Ratio
	(95% CI)	(95% CI)	(95% CI)	(95% CI)	(95% CI)	(95% CI)
Good childhood health	1.99	1.69	1.77	67.19	27.34	22.96
	(1.77–2.24)	(1.51–1.90)	(1.58–1.98)	(36.09–125.08)	(14.80–50.49)	(12.37–42.61)
The residual of the first stage				0.15	0.22	0.25
				(0.11–0.21)	(0.16–0.31)	(0.18–0.35)
Education level (Junior high school or below serves as the reference group)						
Senior high school	0.73	1.52	1.63	0.59	1.29	1.40
	(0.61–0.86)	(1.29–1.79)	(1.38–1.91)	(0.50–0.70)	(1.09–1.52)	(1.18–1.65)
College or above	0.57	1.94	2.55	0.65	2.16	2.82
	(0.30–1.08)	(1.08–3.48)	(1.33–4.88)	(0.34–1.25)	(1.19–3.92)	(1.48–5.36)
Current smoker	0.66	1.98	2.04	0.35	1.17	1.25
	(0.58–0.75)	(1.75–2.24)	(1.80–2.30)	(0.29–0.41)	(0.99–1.38)	(1.06–1.48)
Current drinker	1.08	1.20	1.27	1.06	1.18	1.25
	(0.93–1.24)	(1.04–1.38)	(1.10–1.46)	(0.92–1.22)	(1.03–1.36)	(1.09–1.44)
Not married	0.72	2.22	2.41	0.35	1.23	1.40
	(0.61–0.86)	(1.89–2.61)	(2.04–2.84)	(0.28–0.44)	(1.00–1.51)	(1.13–1.73)
Male	0.97	0.50	0.51	1.27	0.62	0.62
	(0.87–1.07)	(0.45–0.55)	(0.46–0.57)	(1.13–1.42)	(0.55–0.69)	(0.56–0.70)
Aged 45–50	0.53	1.75	1.84	0.91	2.67	2.73
	(0.46–0.60)	(1.52–2.02)	(1.60–2.13)	(0.77–1.08)	(2.27–3.16)	(2.31–3.22)
Aged 50–55	0.27	1.35	1.17	1.26	4.61	3.65
	(0.24–0.30)	(1.20–1.52)	(1.04–1.32)	(0.94–1.70)	(3.42–6.21)	(2.70–4.93)
Aged 55–60	0.51	1.69	1.62	1.00	2.84	2.62
	(0.41–0.65)	(1.32–2.16)	(1.26–2.08)	(0.77–1.31)	(2.18–3.71)	(2.00–3.44)

**Table A2:** Impact of self-rated health up to and including age 15 on health outcome: results from the first stage (the Logistic model) of the 2-stage Residual Inclusion estimation (2SRI).

Variable	Self-Rated Health Up to and Including Age 15
	Coefficient (95% CI)
The fluctuation of grain production (instrumental variable)	2.95 (2.48–3.42)
Education level (Junior high school or below serves as a reference group)	
Senior high school	0.25 (0.07–0.43)
College or above	−0.25 (−0.92–0.43)
Current smoker	0.73 (0.59–0.87)
Current drinker	−0.03 (−0.18–0.13)
Not married	0.77 (0.59–0.96)
Male	−0.26 (−0.37–0.14)
Aged 45–50	1.04 (0.77–1.30)
Aged 50–55	0.93 (0.62–1.25)
Aged 55–60	−0.32 (−0.58–0.06)
Non-famine cohort	−0.65 (0.84–0.46)
Father’s education level (Junior high school or below serves as a reference group)	
Senior high school	0.28 (−0.12–0.67)
College or above	−0.10 (−0.33–0.13)
The log of number of siblings	−0.18 (−0.31–0.06)
Father lived longer than 65 years	0.27 (0.09–0.45)

Note: Provinces' fixed effects were included. Note on the instrumental variable: the birth year’s fluctuation of grain production in the birth area was positively correlated with childhood health status and was statistically significant at the 1% level.

**Table A3:** Impact of self-rated health up to and including age 15 on health outcome: results from the first stage (the ordinary least-square model) of the 2-stage least-squares estimation (2SLS).

Variable	Self-Rated Health Up to and Including Age 15
	Coefficient (95% CI)
The fluctuation of grain production (instrumental variable)	0.58 (0.50–0.66)
Education level (Junior high school or below serves as a reference group)	
Senior high school	0.04 (0.01–0.07)
College or above	−0.04 (−0.16–0.09)
Current smoker	0.14 (0.11–0.16)
Current drinker	−0.01 (−0.03–0.02)
Aged 45–50	0.18 (0.14–0.23)
Aged 50–55	0.18 (0.14–0.23)
Aged 55–60	−0.09 (−0.14–0.04)
Non-famine cohort	−0.12 (−0.16–0.09)
Male	−0.05 (−0.07–0.03)
Not married	0.15 (0.12–0.18)
Number of sibling	−0.03 (−0.06–0.01)
Father lived longer than 65 years	0.05 (0.02–0.08)
Father’s education level (Junior high school or below serves as a reference group)	
Senior high school	0.04 (−0.02–0.11)
College or above	−0.02 (−0.06–0.02)
Test of excluded instruments	
F statistic	183.88
Under-identification tests	
Anderson canon. corr. LM statistic	180.57
Weak identification test	
Cragg–Donald Wald F statistic	183.88
Weak-instrument robust inference	
Anderson–Rubin Wald test: F statistic	207.80
Anderson–Rubin Wald test: Chi-square statistic	209.04
Stock-Wright LM *S* statistic	203.43

Notes: Std. errors are robust. This table shows results from the first stage of the two-stage least-squares (2SLS) estimation, as we would like to show the validity of our instrumental variable (even if we were not able to implement the two-stage least-squares estimation due to the binary nature of adult health outcomes in the second stage). The instrumental variable is positively related to childhood health status (*p* < 0.01). The test of the joint significance of the instruments yielded an F-statistic of 247.32 (*p* < 0.01). A number of statistical tests also indicated that our instrument was valid (assessments by the excluded-instrument test, underidentification test, weak-identification test and weak-instrument-robustness test). Provinces' fixed effects were included.
